# Comparative study of physiologic characteristics between the newly compiled Bafa Wubu of tai chi and 24 form simplified tai chi

**DOI:** 10.1186/s13102-020-00192-x

**Published:** 2020-07-29

**Authors:** Shaojun Lyu, Jianwei Zhang, Jianquan Nie, Cuihan Li, Tianming Gao, Wen Yuan, Zaihao Chen, Jing Ma

**Affiliations:** 1 College of Physical Education and Sports, Beijing Normal University, Beijing, 100875 China; 2 Party Office, The Central Institute of Ethnic Administrators, Beijing, 100094 China; 3 College of Wushu, Beijing Sport University, Beijing, 100084 China; 4 Department of Cardiology, First Medical Center of Chinese People’s Libration Army General Hospital, Beijing, 100853 China

**Keywords:** Bafa Wubu of tai chi, 24 form simplified tai chi, Energy metabolism, Exercise intensity, Oxygen uptake

## Abstract

**Background:**

The newly compiled Bafa Wubu of Tai Chi (Eight Methods and Five Footworks) is a fitness routine that has been developed in accordance with the appeal of the General Administration of Sport of China and promoted both in China and abroad. This paper aims to compare the differences in energy consumption and related parameters between the two types of Tai Chi.

**Methods:**

A total of 60 healthy participants were recruited; 37 males (aged 37.4 ± 10.4 years) and 23 females (aged 31.9 ± 12.8 years). The maximal exercise capacity of participants was measured at baseline. Then, they received Tai Chi training for 12-week and their energy metabolism was measured dynamically.

**Results:**

A set of the Bafa Wubu of Tai Chi requires approximately 3 min, while a set of 24 form simplified Tai Chi approximately 5 min and 40 s. The average oxygen uptake/kg (VO_2_/kg, 10.8 ± 2.52 ml/kg/min vs. 12.9 ± 2.59 ml/kg/min, *P* = 0.000), the highest VO_2_/kg (19.3 ± 6.03 ml/kg/min vs. 24.1 ± 7.50 ml/kg/min, *p* = 0.000, the average metabolic equivalent (METs,2.3 ± 0.16 METs vs. 3.2 ± 0.14 METs, *p* = 0.000), the highest oxygen pulse (VO_2_/HR, 11.1 ± 0.99 ml vs. 13.9 ± 0.93 ml, *p* = 0.000) and rate of perceived exertion (RPE, 10.7 ± 0.70 vs. 1.3 ± 0.62, *p* = 0.000) gained immediately after Bafa Wubu of Tai Chi exercise were significantly lower than those in 24 form simplified Tai Chi; heart rate recovery (HRR,1.5 ± 0.41 vs. 1.3 ± 0.45, *p* = 0.008) at 1 min after the practice was significantly higher than after the 24 form simplified Tai Chi. Meanwhile, the average heart rate (HR, 104.1 ± 11.41 bpm vs. 105.7 ± 9.68 bpm, *p* = 0.696) and the highest respiratory quotient (RQ, 1.0 ± 0.06 vs. 0.9 ± 0.09, *p* = 0.643) were not significantly different. The intensity of Tai Chi was described as the highest oxygen uptake of the participants when they performed the Tai Chi divided by their individual maximal oxygen uptake. Tai Chi intensity during Bafa Wubu of Tai Chi (50% ± 11.7% vs. 64% ± 12.5%) was significantly lower than during 24 form simplified Tai Chi.

**Conclusion:**

The newly compiled Bafa Wubu of Tai Chi is characterized by lower energy consumption than 24 form simplified Tai Chi.

**Trial registration:**

Ethics Committee of Sports Science Experiment, Beijing Sport University- 2018010H. Registered 19 June 2018

## Background

Studies have shown that Tai Chi, a type of traditional Chinese martial arts, improves muscle strength [1–4], physical balance [5–7], cardiopulmonary function [8–10] and mental health [11, 12]. In recent years, the research of its effects on rehabilitation for patients with chronic diseases has also attracted the attention of domestic and foreign scholars [13–18]. A study by Zhou [19] showed that the risk of losing muscle strength and falling down in 205 elderly long-term Tai Chi practitioners was significantly lower than those in the control group without Tai Chi experience. Sato’s [20] study demonstrated that 12 months of Tai Chi practice combined with conventional exercise training improved the reflex function of the vagus nerve in patients with coronary heart disease. Another study [21] indicated that 12 weeks of Tai Chi practice improved BMI, blood lipids, C-reactive protein levels and malondialdehydes in patients with diabetes mellitus. Tai Chi was also found to reduce pain, enhance physical immunity and improve sleep quality in patients with ischemic stroke [22].

Of the many styles of Tai Chi, the 24 form simplified Tai Chi, which was created by the General Administration of Sport of China in 1956; however, it is hard to further popularize and generalize, due to its numerous movements and complexity. Therefore, our team, at the request of the General Administration of Sport of China, developed the newly compiled Bafa Wubu of Tai Chi, namely, introductory routines to Tai Chi characterized by simple structures, and has promoted its application domestically and internationally since July 2018. There are some differences in some postures between these two Tai Chi styles. Until now, the differences in physiology between the newly compiled Bafa Wubu of Tai Chi and 24 form simplified Tai Chi had never been studied. We hypothesized that the newly compiled Bafa Wubu of Tai Chi exhibits lower energy consumption than 24 form simplified Tai Chi. Therefore, this study mainly compared the differences in physiological characteristics of the two styles of Tai Chi.

## Methods

### Study design

**Fig. 1:**
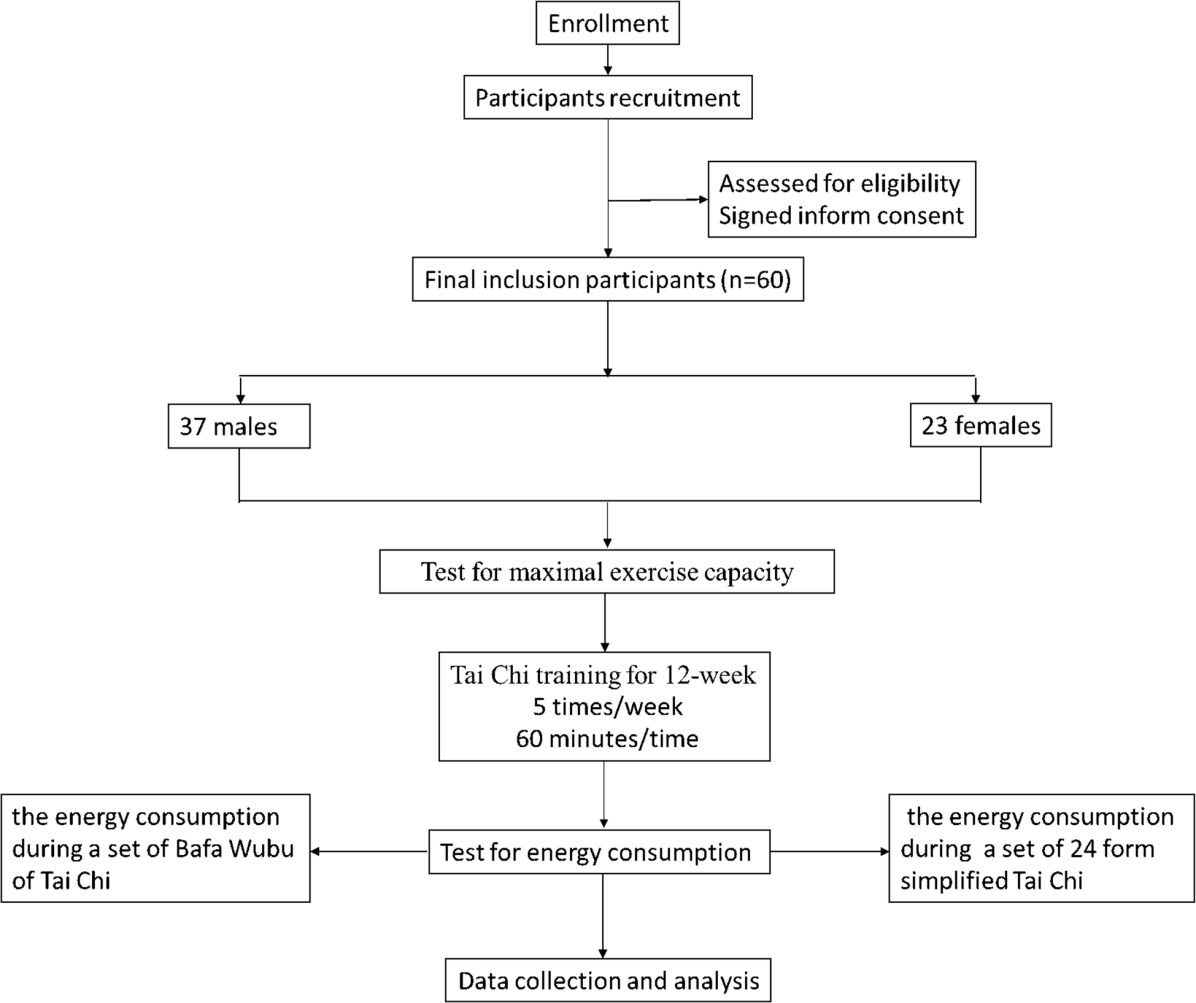
Flow diagram of study design

The maximal exercise capacity of all the participants was measured at baseline. Then, they received Tai Chi training for 12-week to ensure they all mastered both Bafa Wubu of Tai Chi and 24 form simplified Tai Chi. When the participants practiced a set of the newly compiled Bafa Wubu of Tai Chi and 24 form simplified Tai Chi, their energy metabolism was measured dynamically. A brief flowchart of the entire study is shown in Fig. 1. This study was approved by the Ethics Committee of Sports Science Experiment of Beijing Sport University (2018010H).

### Participants

A total of 60 healthy participants (37 males aged 37 ± 10.44 and 23 females aged 32 ± 12.75) were recruited from Beijing Normal University and the Central Institute of Ethnic Administrators. Inclusion criteria were as follows: ①age of the participants was limited to between 20 and 60; ② participants were interested in Tai Chi and were Tai Chi beginners, practicing Tai Chi for less than 1 month; ③participants were healthy with no metabolic diseases; ④participants signed the Informed Consent Form. In addition, those with movement dysfunction and long-term Tai Chi practitioners were excluded from the study.

### Test for maximal exercise capacity

The participants received a physical examination to collect information about height, weight, blood pressure and oxygen saturation at baseline. High-intensity exercise, smoking, alcohol and coffee were not allowed for at least 24 h before the test. Test for maximal exercise capacity was performed at 1 hour after meal. Then, their exercise capacities were measured following the Bruce Treadmill protocol on the cardiopulmonary exercise test machine (CORTEX Metal2, Germany). The load protocol starts at 2.7 miles per hour and a gradient of 10%, with an increase of 2% gradient and 1.3 miles per hour of speed every 3 min.

### Plans for tai chi guidance

After the cardiopulmonary exercise test, all participants received Tai Chi training involved both the newly compiled Bafa Wubu of Tai Chi and 24 form simplified Tai Chi for 12 weeks, from June to August 2018. The whole Tai Chi training was directed by professional Tai Chi instructors. Tai Chi training class was held five times a week. One class requires 60 min, consisting of 10 min of warm-up activities, 40 min of practice and 10 min of closing activities. Not only the postures but also the combination of body, breath and mind were emphasized. Tai Chi exercise contains three core elements, namely “body”, “breath” and “mind”. The spirits of Tai Chi are summarized to “building body”, “conveying breath” and “using mind”. Once the training was finished, Tai Chi assessment experts conducted a test to check whether the participants had mastered both Tai Chi styles. To guarantee the study quality, the main investigators, Tai Chi instructors and assessment experts were different persons and independent to each other.

### Test for energy consumption

Devices should be calibrated before tests. Generally, device vector table (DVT) and ambient air are calibrated once before each test (once a day). After Tai Chi training, the energy consumption during a set of the newly compiled Bafa Wubu of Tai Chi or 24 form simplified Tai Chi were tested by the MateMax3B portable cardiopulmonary tester (Cortex Gas Metabolizer, German Cortex), which could output indices such as oxygen uptake/kg (VO_2_/kg), metabolic equivalent (METs), heart rate (HR), heart rate recovery (HRR), oxygen pulse (VO_2_/HR) and respiratory quotient (RQ). The heart rate recovery within 1 minute after the ending of Tai Chi practice was monitored and recorded by MateMax3B tracking application. The Inbody3.0 body composition analyzer produced by BIOSPACE in South Korea was used to measure the height, weight, body fat ratio, fat-free mass (FFM) and body mass index (BMI).

Participants avoided high-intensity exercise, smoking, alcohol and coffee for at least 24 h prior to when the energy consumption test was performed at 1 hour after meal. Firstly, devices should be calibrated before tests. After calibrating the ambient gas pressure and gas flow rate, the participants wore the Mate Max 3B testing mask. The process started with quiet sitting for 10 min, followed by a rise to the Starting Posture by following the Tai Chi music and then proceeded through the set of Tai Chi exercises. Physiological indices, including HR, blood pressure, oxygen saturation, VO_2_max/kg, METs, HR, VO_2_/HR and RQ, were measured dynamically. The newly compiled Bafa Wubu of Tai Chi was tested first, and the 24 form simplified Tai Chi was tested 2 days later. Each participant was tested three times at least 1 hour apart. Finally, the data from the three tests were averaged as the final result.

### Statistical analysis

The measured data were processed with SPSS19.0 statistical software, with a sampling frequency of 60 times/min. The indices are presented by the average value ± the standard deviation with the unit of seconds; all the average values were tested by paired t-tests, with a *p* < 0.05 regarded as statistically significant, and a *p* < 0.01 regarded as substantial statistical significance.

## Results

### General characteristics

The study recruited a total of 60 participants, including 37 males (aged 37 years ±10.4) and 23 females (aged 32 years ±12.8). The average BMI was 23.4 ± 2.85 kg/m^2^, the average body fat ratio was 21.9 ± 5.27, and the average FFM mas was 52.5 ± 8.99 kg. We performed the cardiopulmonary exercise test to investigate the maximal exercise capacity of the participants before they learned Tai Chi. The results are described in Table 1.

**Table 1:** Maximal exercise capacity of participants

n	VO_2_max/kg (ml/kg/min)	METsmax (METs)	HRmax (bpm)	VO_2_/HRmax (ml)	RQmax	RPEmax
60	40.6 ± 5.86	12.2 ± 2.11	185 ± 10.4	16.2 ± 1.31	1.23 ± 0.154	18.2 ± 0.07

Data presented are Mean SD. *METsmax* the maximal metabolic equivalent, *HRmax* the maximal heart rate, *VO*_*2*_*/HRmax* the maximal pulse oxygen, *RQmax* the maximal respiratory quotient, *RPEmax* the maximal rate of perceived exertion

### Physiologic characteristics of the participants at rest

The participants sat quietly for 10 min before the test and then wore breathing masks to have their physiological characteristics tested before the practice of Tai Chi. This baseline information is shown in Table 2.

**Table 2:** Basic characteristics of participants in the quiet state before the test

	n	Before Bafa Wubu of Tai Chi	Before 24 form simplified Tai Chi	t	P
Systolic Pressure	60	111.8 ± 6.18	113.2 ± 6.00	−0.492	0.142
Diastolic Pressure	60	65.1 ± 7.01	66.2 ± 6.61	−0.574	0.568
Oxygen Saturation	60	96.9 ± 1.03	97.1 ± 1.29	−1.202	0.235
VO_2_/kg	60	7.1 ± 2.73	7.4 ± 2.89	−1.200	0.335
METs	60	1.1 ± 0.17	1.1 ± 0.22	0.440	0.662
HR	60	78.6 ± 5.43	78.1 ± 3.51	0.653	0.516
VO_2_/HR	60	5.7 ± 2.53	5.6 ± 2.11	0.114	0.910
RQ	60	0.7 ± 0.08	0.7 ± 0.10	0.209	0.835

Data presented are Mean SD. METs, metabolic equivalent; HR, heart rate; VO2/HR, oxygen pulse; RQ, respiratory quotient

The basic data of the participants before the test is shown in Table 2. The results show that there was no significant difference in the physiological indexes of the participants before the practice of the newly compiled Bafa Wubu of Tai Chi and 24 form simplified Tai Chi, suggesting the next tests were comparable.

### Comparison of physiological indexes after the practice of tai chi

The participants completed both a set of the newly compiled Bafa Wubu of Tai Chi and 24 form simplified Tai Chi and the physiological characteristics after each, including VO_2_/kg, HR, HRR, VO_2_/HR, RQ and blood pressure, were compared.

As shown in Table 3, during the practice of Bafa Wubu of Tai Chi, the values of average VO_2_/kg (10.8 ± 2.52 ml/kg/min vs. 12.9 ± 2.59 ml/kg/min), the highest VO_2_/kg (19.3 ± 6.03 ml/kg/min vs. 24.1 ± 7.50 ml/kg/min), and the highest VO_2_/HR (11.1 ± 0.99 ml vs. 13.9 ± 0.93 ml) were significantly smaller than those in the 24 form simplified Tai Chi, with *P* < 0.01. The HRR (1.47 ± 0.41 vs. 1.27 ± 0.45) after the practice of the newly compiled Bafa Wubu of Tai Chi was significantly higher than after the practice of 24 form simplified Tai Chi, with *P* = 0.008. During the practice process of the newly compiled Bafa Wubu of Tai Chi, there were no significant differences in the values of average HR, highest RQ, systolic pressure, diastolic pressure or oxygen saturation obtained immediately after exercise compared with those in the 24 form simplified Tai Chi.

**Table 3:** Comparisons of Bafa Wubu of Tai Chi vs. 24 form simplified Tai Chi in physiologic indices

	n	The newly compiled Bafa Wubu of Tai Chi	24 form simplified Tai Chi	t	P
Average VO_2_/kg	60	10.8 ± 2.52	12.9 ± 2.59	−4.877	0.000
Highest VO_2_/kg	60	19.3 ± 6.03	24.1 ± 7.50	−4.030	0.000
Average HR	60	104.1 ± 11.41	105.7 ± 9.68	−0.393	0.696
HRR	60	1.47 ± 0.41	1.27 ± 0.45	2.775	0.008
Highest VO_2_/HR	60	11.1 ± 0.99	13.9 ± 0.93	−14.193	0.000
Highest RQ	60	1.0 ± 0.06	0.9 ± 0.09	0.466	0.643
Systolic Pressure Obtained Immediately after Exercise	60	134.5 ± 8.66	136.2 ± 4.94	−1.276	0.207
Diastolic Pressure Obtained Immediately after Exercise	60	69.6 ± 6.41	69.2 ± 7.90	−0.095	0.925
Oxygen saturation Obtained Immediately after Exercise	60	97.1 ± 1.30	97.2 ± 1.21	−0.406	0.686

Data presented are Mean SD. *HR* heart rate, *HRR* heart rate recovery, *VO*_*2*_*/HR* oxygen pulse, *RQ* respiratory quotient

### Comparison of tai chi intensity in the practice of Bafa Wubu of tai chi and 24 form simplified tai chi

To further analyze the intensity of the newly compiled Bafa Wubu of Tai Chi vs. 24 form simplified Tai Chi, we compared the average METs, RPE and the ratios of the highest oxygen uptake of the participants when they performed the Tai Chi over their individual maximal oxygen uptake. The results are described in Table 4.

**Table 4:** Comparison of Bafa Wubu of Tai Chi vs. 24 Form Simplified Tai Chi in Tai Chi intensity

	n	The newly compiled Bafa Wubu of Tai Chi	24 form simplified Tai Chi	t	P
Tai Chi intensity	60	0.50 ± 0.117	0.64 ± 0.125	−10.652	0.000
Average METs	60	2.3 ± 0.16	3.2 ± 0.14	−29.742	0.000
Average RPE	60	10.7 ± 0.70	11.3 ± 0.62	−4.968	0.000

Data presented are Mean SD. *METs* metabolic equivalent, *RPE* rate of perceived exertion

Oxygen uptake during one set of Bafa Wubu of Tai Chi occupied 50% ± 11.7% of their individual maximal oxygen uptake, which was significantly lower than that of 24 form simplified Tai Chi (64% ± 12.5%), *p* = 0.000. Accordingly, the average METs of Bafa Wubu of Tai Chi were 2.3 ± 0.16 METs, which was much lower than that of 24 form simplified Tai Chi (3.2 ± 0.14 METs), *p* = 0.000. The RPE gained immediately after Bafa Wubu of Tai Chi exercise was 10.7 ± 0.70, which was much lower than RPE after 24 form simplified Tai Chi (11.3 ± 0.62), *p* = 0.000.

## Discussion

The newly compiled Bafa Wubu of Tai Chi is the latest introductory routine introduced by the General Administration of Sport of China in 2018; it has been promoted and applied in China and abroad since July 2018. Our study compared, for the first time, the differences in physiological characteristics between the newly compiled Bafa Wubu of Tai Chi and 24 form simplified Tai Chi.

During the practice of the newly compiled Bafa Wubu of Tai Chi, the values of average VO_2_/kg, the highest VO_2_/kg, the average METs, the maximum VO_2_/HR, and the RPE obtained immediately after exercise and Tai Chi intensity were all significantly smaller than those in the 24 form simplified Tai Chi. Meanwhile, heart rate recovery after the practice of Bafa Wubu of Tai Chi was significantly higher than after 24 form simplified Tai Chi. The energy metabolism analysis demonstrated that the newly compiled Bafa Wubu of Tai Chi belonged to the low-intensity exercise category, which is lower than 24 form simplified Tai Chi.

Our study reported, for the first time, that the average VO_2_/kg was 10.8 ± 2.52 ml/kg/min and the highest VO_2_/kg was 19.3 ± 6.03 ml/kg/min during a set of the newly compiled Bafa Wubu of Tai Chi, which were both much lower than those during 24 form simplified Tai Chi. Pate RR [23] invented a model to identify the intensity of exercise by average METs. In his model, an exercise whose average METs was lower than 3 was stratified to be low intensity. Meanwhile, exercises with average METs between 3 and 6 were medium intensity. According to this model, the average METs of a set of the newly compiled Bafa Wubu was 2.3 ± 0.16, indicating it should be categorized as low intensity. The average METs of a set of 24 form simplified Tai Chi was found to be 3.2 ± 0.14 in our study, placing it in the medium intensity category. Chao’s [24] group also reported an average of 3 METs during the practice of 24 form simplified Tai Chi, which is similar to the results of our study. We also analyzed the intensity by the ratio of highest VO_2_/kg over individual peak VO_2_/kg。The intensity after a set of the newly compiled Bafa Wubu of Tai Chi was 50%, which was much lower than the value of 64% after 24 form simplified Tai Chi. Our study revealed that the intensity of the newly compiled Bafa Wubu of Tai Chi is much lower than 24 form simplified Tai Chi. In this study, the RPE obtained immediately after practicing the newly compiled Bafa Wubu of Tai Chi was 10.7 ± 0.70, while the RPE obtained immediately after the practice of the 24 form simplified Tai Chi was 11.3 ± 0.62, which means practicing the newly compiled Bafa Wubu of Tai Chi was much more relaxing than practicing 24 form simplified Tai Chi.

It is reported that the recovery of heart rate 1 min after exercise has been routinely used in studies, which is an important parameter to reflect the degree of heart health [25, 26]. Heart rate recovery is independent of workload or age, blunted with left heart failure, and accelerated in athletes [27]. It is characterized by a dynamic interplay between parasympathetic reactivation and sympathetic withdrawal and has been recognized as a noninvasive but feasible measure of autonomic function [28, 29]. HRR has also being used as a valuable tool to monitor changes in physical performance and training status in athletes in recent years [30, 31]. The higher the HRR, the faster your cardiac recovery, which means your heart is healthier to some degree. Although there was no significant difference in the average HR and the highest HR between the newly compiled Bafa Wubu of Tai Chi and the 24 form simplified Tai Chi, our study showed that the heart rate decreased much more significantly within 1 minute after the practice of Bafa Wubu of Tai Chi than after the practice of 24 form simplified Tai Chi (1.47 ± 0.41 vs. 1.27 ± 0.45, *p* = 0.008). Better heart recovery suggests that participants of the practice of Bafa Wubu of Tai Chi exhibited better regulating function of the vagal nerve.

Zhuo, D [32] found that the average METs during the practice of Tai Chi was 4.1, which was slightly higher than in our study. We thought the reason might be that his study adopted 48 Form Tai Chi, which lasts a longer time and has more movements compared with both the newly compiled Bafa Wubu of Tai Chi and 24 form simplified Tai Chi. It is reported, according to Young, DR [33] that the average HR during the practice of Yang-style Tai Chi was approximately 116 bpm, slightly higher than during our 24 form simplified Tai Chi. We thought the reason might be that Young, DR adopted a low-frame Tai Chi practice, while the high-frame Tai Chi practice was employed in our study, which shows that the Tai Chi frame directly affects the intensity of Tai Chi practice. It shows that the time consumption and the height of frame of Tai Chi maybe have effects on physiologic indices.

We further analyzed the reason why the newly compiled Bafa Wubu of Tai Chi differed significantly from 24 form simplified Tai Chi. The reasons might be attributable to the following factors. Firstly, the postures of the newly compiled Bafa Wubu of Tai Chi differs from 24 form simplified Tai Chi. The newly compiled Bafa Wubu of Tai Chi is further simplified from 24 form simplified Tai Chi. Compared with the 24 form simplified Tai Chi, the newly compiled Bafa Wubu of Tai Chi boasts a simple structure of movements, a reasonable number of postures, easy-to-learn features, fewer practice environment limitations. Secondly, the movement trail of the newly compiled Bafa Wubu of Tai Chi differs from 24 form simplified Tai Chi. The newly compiled Bafa Wubu of Tai Chi has two stages. The first stage is to practice the first 17 postures for the in situ exercise of staying, requiring that the upper limbs employ the eight hand techniques while the lower limbs are fixed. The second stage is to practice the last 15 postures, where the hand techniques are combined with footwork for marching exercises. Comparatively, the 24 form simplified Tai Chi keeps the marching exercises, with the combination of hand techniques and footwork, throughout. Finally, a set of the newly compiled Bafa Wubu of Tai Chi takes less time (approximately 3 min) than 24 form simplified Tai Chi (5 min and 40 s), suggesting the physical difference might partially be influenced by the different time consumption.

## Conclusion

Our study found that the newly compiled Bafa Wubu of Tai Chi is characterized by lower energy consumption than 24 form simplified Tai Chi.

## Data Availability

The datasets used and analysed during the current study are available from the corresponding author on reasonable request.

## Abbreviations

HR: Heart rate
HRR: Heart rate recovery
RQ: Respiratory quotient
FFM: Fat-free mass
BMI: Body mass index
DVT: Device vector table
